# Acceptance of COVID-19 vaccine and determinant factors in the Iranian population: a web-based study

**DOI:** 10.1186/s12913-022-07948-w

**Published:** 2022-05-16

**Authors:** Shabnam Omidvar, Mojgan Firouzbakht

**Affiliations:** 1 Social Determinants of Health Research Center, Health Research Institute, Babol University of Medical Sciences, Babol, IR Iran; 2 Department of Nursing- Midwifery, Comprehensive Health Research Center, Babol Branch, Isalamic Azad University, Babol, Iran

**Keywords:** Hesitancy, COVID-19, Vaccination, Iran

## Abstract

**Background:**

Iran had a high rate of death in several COVID 19 waves. Vaccination is a method for prevention and control of the COVID-19 pandemic. Success in controlling the pandemic is not solely dependent on the effectiveness of the vaccines. It is also dependent on the global acceptance and vaccine coverage rate. This study aimed to determine the acceptance rate of COVID-19 vaccination in the Iranian population and the factors affecting it.

**Methods and materials:**

This study was a cross-sectional research on 1564 Iranian people above 18. Study data were collected using a web-based questionnaire and analyzed using linear regression analysis and logistics at a significance level of 0.05 using SPSS.

**Results:**

Approximately 70% of the participants reported acceptance of the vaccines. Ten percent of the people were against and 20% were hesitant to get vaccinated. The results showed that risk perception (*P* = .003), Knowledge of the disease (*P* < .001), trust in the health system (*P* < .001), attitude towards vaccination (P < .001), and vaccination literacy (P < .001) were predictors of vaccine acceptance. People with higher levels of education and mistrust towards the health system had a reduced vaccine acceptance rate.

**Conclusions:**

The acceptance rate of the COVID-19 vaccine in Iran was higher than in the other countries in the Middle East. Extensive interventions are important to increase the trust in the health system and improve the knowledge of vaccine efficacy and literacy.

## Introduction

The pandemic COVID-19 has been reported in over 144 countries worldwide. This viral disease has infected over 238 million and has taken 4,859,596 lives, up to date (9th October 2021) [1]. Over 5 million infections were reported among the Iranian population, out of which, almost 123,000 patients passed away due to the severe symptoms of COVID-19 (9th October 2021) [2]. Long-term success in encountering this outbreak is believed to be herd immunity, which suggests that 67% of the population must become immune to this disease. For such a large population to be infected, live through and become immune to COVID-19, it is estimated that more than 30 million people will lose their lives. In addition to that, infection of such a large ratio of the world’s population with COVID-19 will exert extreme pressure on the medical community [3].

In addition to the obvious effects of this disease on global health, it has also proven to be taxing to the economic and social status of the people [4, 5]. The most important intervention against COVID-19 is believed to be mass vaccination, encouraging hundreds of organizations and institutions to strive for high-efficacy vaccine production [6–12]. It has been reported that multiple factors contribute to the acceptance rate of new vaccines [10, 13–15]. These factors include the efficacy of the vaccine, adverse effects, misinformation, etc.; which can affect the public acceptance of vaccines and pose risk to public health [15, 16].

During the pandemic influenza, the acceptance rate of vaccines was reported to range from 8 to 67% [17]. This rate was reported to be 64% in the US [13]. In a study in the UK, 56.1% of the participants accepted the H1N1 vaccines [18]. Similarly, another research in Beijing reported a 59.5% acceptance of the vaccine against H7N9 in the future [9]. The acceptance of vaccines is a complex context and is highly affected by time, location, as well as the social behavior of the society [10–15, 17]. A study in Ireland reported that healthcare personnel avoided the seasonal influenza vaccine due to misinformation, inefficacy assumption and mistrust of the vaccine [16]. A research in China, announced demographic characteristics and the understanding of the society toward the vaccine, as acceptance predictors [9]. Anxiety level and the history of vaccinations were the predictors reported by a study in Hong Kong [18]. Another study in the US suggests that the social effects and insurance are key predictors of vaccine acceptance [19]. A study on the opinion of parents on children’s vaccination in the UAE reported that 12% of parents do not trust vaccines. It was concluded that immunity, side effects and multiple injections were the main factors for the doubts [20]. Although people with a history of vaccination against seasonal flu reported beingwilling to receive H1N1 vaccines [14, 15], a study in April 2020 reported that 26% of the adults in 7 European countries were unwilling to receive vaccination [21]. Similar studies suggest that almost one-fourth of the population of France [22] and the US [23] were also unwilling to receive the vaccination. Studies at the end of July (when restrictions were reduced) report that even more people were unwilling to receive vaccination in the UK [24]. In these studies, women [21, 22, 25–27], people with lower education [22, 24–26], people with less precautious measures against COVID19 [22] and people who did not receive the seasonal flu vaccine in the passing year were more likely to refuse vaccination [23, 28].

As COVID19 is a novel virus, there are only limited numbers of studies on acceptance of vaccines and attitudes towards COVID 19 vaccines [23, 29]. Because the acceptance of vaccines follows many different factors, therefore, the acceptance rate of vaccination in different communities is unpredictable and also there has not been such a study among the Iranian population regarding the acceptance of the COVID19 vaccines, then we decided to conduct this study.

## Methods and materials

### Study design

The study was a cross-sectional web-based investigation that was conducted in May 2021 in Iran. The sampling was conducted during the fourth wave of the COVID-19 outbreak in Iran.

### Inclusion criteria

Iranian nationality, above 18 years, the literacy to read and write, access to smartphones, computers, or tablets, and being members of social media.

### Exclusion criteria

People who were not willing to participate in the study.

The data collection was done via the Snow Ball method where the questionnaire was initially distributed on social media and the receivers were requested to share the questionnaire in their group chats. This survey was released on the website https://survey.porsline.ir/s/aymzC7b .

### Ethics approval and consent to participate

The study was approved by the Research Ethical Committee of Babol University of Medical Sciences (IR.MUBABOL.REC.1400.081). Written consent was taken from all the participants. Filling out the questionnaire was voluntary and the written consent form and all the explanations were displayed on the first page of the questionnaire; right before the main questionnaire.

### Determination of the sample size

The results from similar studies indicate that 15% of the participants were hesitant about the COVID19 vaccination [1]; hence, considering a reliability factor of 0.95, the ratio of .020 and an acceptable error of 0.2, the sample size was determined to be 1500 according to the equation below.

$$n=\frac{z_{1-\frac{\alpha }{2}}^2\ p\left(1-p\right)}{d^2}$$

### Instruments

The mentioned survey included the following questionnaire:

1. Demographic questionnaire: consisting of age (Year), gender (Female/Male), marital status (Married/single), education (High school/Diploma/Undergraduate/Graduate), and residence (Urban/Rural) information.
2. Risk perception of COVID19 questionnaire. The questionnaire consisted of 4 questions that were answered as yes/no. These questions included: - Do you consider yourself at risk for COVID-19? Have you ever been infected with COVID-19? Have any of your relatives been contaminated by COVID-19? Have any of your relatives passed away due to COVID-19?
3. Knowledge and precautions against COVID19 questionnaire.

The information from previous research [30] and the protocols presented by the WHO and the Iranian Ministry of Health inspired this original questionnaire. The first 13 questions were on knowledge, assessing the knowledge ofprecautious measures, infection methods and control methods for COVID19. The scoring for this section was through choice analysis (yes = 1, no & I do not know = 0) summing up a maximum of 13 and a minimum of zero in the knowledge section. It was assumed that participants with at least 70% of the maximum score possess acceptable knowledge on the matter. The questions related to the view on the Pandemic were whether they think that the pandemic can be controlled and whether they think that they will successfully control the pandemic. The scoring was similar to the previous section with a maximum score of 2 and a minimum of zero. The other set of questions (3 questions) focused on preventive behavior such as the utilization of masks and gloves, as well as whether the participants go to gatherings. This set of questions was also assessed in the same way as the ones mentioned before and the maximum and minimum scores were 3 and zero, respectively.

4. Attitude towards vaccine questionnaire.

This questionnaire included 12 questions [31] with a spectrum of scores ranging from 1 (strongly agree) to 6 (strongly disagree) with 4 subscales: 1) Lack of trust invaccine proficiency 2) Lack of trust to the unknown side effects in the future 3) Lack of trust inthe financial use of vaccines and 4) preference of natural immunity. The validity and reliability of the questionnaire werestudied in two studies in the US and the UK (Alpha Cronbach = 0.77 ~ 0.93) [32, 33].

5. Vaccine literacy questionnaire

This self-reported questionnaire was developed by Ishikawi to study non-infective chronic diseases [34, 35]. It includes 12 questions and is defined based on Nutbeam’s definition [36]. Out of these 12 questions, 4 questions regarding functional literacy and 8 questions regarding interactive-critical vaccine literacy. The scoring was a 4 score Likert (4 signifies never and 1 signifies often). A higher score signifies higher literacy on vaccines [37].

6. Belief and acceptance towards COVID 19 vaccine questionnaire. The questions and possible answers to this questionnaire are presented in Table 1.

**Table 1:** Belief and COVID-19 vaccine acceptance among participants (*N* = 1564)

Variables	Yes *n* (%)	No *n* (%)	Unsure *n* (%)
**Would you get vaccinated?**	1096 (70.1)	152 (9.7)	316(20.2)
**Do you recommend others to get vaccinated?**	1104(70.6)	144(9.2)	316(20.2)
**Do you trust the health system?**	1143(73.1)	421(26.9)	
**Do you trust the vaccine producers?**	1030(65.9)	534(34.1)	
**Would you pay to get vaccinated?**	751(48)	813(52)	
**Have you received the previous flu vaccine**	222(14.2)	1342(85.8)	

Face and content validity assessments were employed to investigate the validity of the questionnaire. Twelve experts rated the questions, and the content validity indices (CVI) were calculated to be 0.81–1. The Cronbach alpha of the questionnaire was 0.79.

### Data analysis

The statistical analysis package SPSS version 21 was employed to analyze the data using descriptive tests and logistic regression with a significance level of 0.05.

## Results

The present study included participants of age between 18 and 81. The mean age of the participants was 37.45 ± 12.37, out of which, 70% were female and 30% were male. Over half of the participants were married and 82% had received college or higher education and 95% were living in urban areas.

Table 1 displays the statistics related to the belief and acceptance of COVID19 vaccines, presenting that almost 30% of the participants were resistant or hesitant regarding the vaccine, almost 27% reported not trusting the health system and 34% reported not trusting the vaccine producers. Over half of the participants were against paying for vaccination and almost 86% of the participants did not receive the influenza vaccine in the previous year.

Among the group who intended to receive the vaccine (1096 participants), 11.4% were interested in the vaccines produced nationally, 42.6% preferred vaccines produced abroad and 46% did not report a preference.

Table 2 exhibits the knowledge, attitude and practice scores. The mean knowledge, attitude and practice scores were rather low, contrary to the vaccine literacy score which was quite high.

**Table 2:** Descriptive statistics of the independent variables (N = 1564)

Variable	Mean (SD)	Min- Max
Knowledge regarding COVID 19	11.09 ± 2.75	8–32
Attitude regarding COVID 19	3.92 ± 1.49	2–6
Practice regarding COVID 19	4.53 ± 0.599	3–6
Attitude towards vaccines	42.25 ± 6.14	12–72
Vaccine literacy	37.77 ± 5.11	12–48

The present study shows a significant relationship between some demographic variables and attitude, knowledge of the disease as well as vaccine literacy. Gender was witnessed to be in a significant relationship with the knowledge of the disease and attitude and literacy of the vaccine. Marital status was also seen to exhibit a significant relationship with the knowledge of the disease and attitude towards the vaccine. A similar relationship was seen between age and attitude as well as vaccine literacy. Education displayed a significant relationship with the three variables mentioned above (Table 3).

**Table 3:** Mean and SD of the study parameters with regard to demographic characteristics

variable		N	knowledge Mean(SD)	T/F	*P* value	Vaccine attitude Mean(SD)	T/F	*P* value	Literacy Mean(SD)	T/F	*P* value
Age	< 30	731	11.21(2.73)	1.49	.226	4.04(1.47)	6.076	.002	34.17(5.04)	10.015	.000
	30–45	603	10.95(2.84)			3.76(1.49)			35.38(5.18)		
	> 45	230	11.06(2.56)			3.97(1.54)			35.10(4.93)		
Gender	Female	1093	10.97(2.6)	−2.57	0.010	42.53(6.02)	2.688	0.007	34.85(5.07)	.946	.344
	Male	471	11.36(3.06)			41.62(6.37)			34.59(5.20)		
Marital status	Single	537	11.42(2.94)	3.39	0.001	42.76(5.82	2.41	0.016	34.43(5.04)	−1.931	.054
	Married	1027	10.92(2.63)			41.99(6.29)			34.95(5.14)		
Residency	Rural	1486	11.07(2.75)	−1.66	0.096	42.28(6.16)	.741	0.459	34.86(5.11)	3.024	.003
	Urban	78	11.60(2.67)			41.75(5.80)			33.07(4.78)		
Education	High school	34	13.14(4.38)		.000	37.79(6.48)	12.61	.000	31.55**(**6.67)		.000
	Diploma	246	11.61**(**2.85)	12.84		40.94(6.21)			33.97(5.54)	22.123	
	Undergraduate	759	11.09(2.71)			42.37(5.77)			34.26(5.02)		
	Graduate	525	10.71(2.52)			42.99(6.4)			36.12(4.58)		

According to the results presented by the Chi-square test, gender was the only demographic characteristic that exhibited a significant relationship with trusting the health system. Female participants trusted the health system more than male participants (*P* = 0.03).

Linear regression analysis was employed to study the predictors of vaccine acceptance. The first model included demographic characteristics as well as the other variables. The second model received the variables in a stepwise manner. The results of the linear regression analysis are displayed in Table 4. In the final model, the risk perception, health system trust, knowledge of COVID19, attitude and literacy of COVID19 vaccines were predictors of vaccine acceptance.

**Table 4:** The unadjusted and adjusted coefficients of linear regression analysis for the vaccine acceptance predictors

Variables	Model 1				Model 2			
	B	T	*P*	adjusted R^2^ Un	B	T	*P*	adjusted R^2^ Un
Age	−.002	−1.385	.166	.210				.213
Gender	−.055	−1.376	.169					
Education	.052-	1.174-	.786					
Residency	.073	1.723	.085					
Risk perception	.071	.838	.402		.133	2.991	.003**	
Do you trust the health system?	.154	2.924	.003**		.585	13.981	< 0.001**	
Knowledge regarding COVID19 disease	0593	14.07	< 0.001		.032	4.589	< 0.001**	
Attitude towards vaccine	.033	4.71	< 0.001**		−.020	_-6.629	< 0.001**	
Vaccine literacy	−.020	−6.562	< 0.001**		−.020	−5.286	< 0.001**	

Model1. The independent variables/ predictors were included linear regression

Model2. The stepwise linear regression method

** *P* < 0.001

The results of the logistic regression analysis signify that education level displayed a significant relationship with vaccine acceptance. The acceptance was seen to be inversely related to education level and the participants with a university education were twice as much likely not to take the vaccine, in comparison to the lower education groups. In addition to that, participants with less trust in the health system were 85% less likely to receive vaccination (Table 5).

**Table 5:** The unadjusted and adjusted OR in logistic regression analysis for predictors of COVID-19 vaccine acceptance

Variables	Model 1				Model2			
	B	sig	OR	CI 95%	B	sig	OR	CI 95%
Age								
< 35	.040	.779	1.041	.786–1.378.				
35–50	0.331	.097	.718	.486–1.061				
50≤	–	0.151	1					
Gender (M/F)	.066	.594	.628	.769–1.3				
Education								
High school	.897	.025	2.451	1.120–5.367	.891	.024	2.438	1.126–5.280
Diploma	.510	.007	1.665	1.153–2.405	.510	.006	1.665	1.161–2.389
Undergraduate	.235	.098	1.265	.958–1.671	.236	.089	1.267	.965–1.663
Graduate		.014	1			.012	1	
Marital status (M/S)	−.149	.284	.861	.656–1/131				
Residency (Urban/rural)	−.304	.257	.738	.436–1.248				
Do you trust the health system?	−1.961	.000	.141	.11–.181	−1.951	.000	.142	.182–.111

Model 1: Unadjusted model

Model 2: Adjusted model

Younger participants were 30% less likely to accept vaccination in compare to participants 50 years or older; however, this difference was statistically insignificant.

## Discussion

The present study aimed to investigate the acceptance of COVID19 vaccination among the Iranian population. To our best knowledge, this study is the only study on vaccine acceptance among the Iranian population. The results signify that the vaccine acceptance rate was 70.1%. Other studies from the Middle Eastern countries exhibited 62 to 69% of acceptance rate. Therefore, the acceptance rate in the Iranian population was more than in the other countries [38, 39].

In a systematic study by Sallam, the highest vaccine acceptance (over 90%) was seen in Ecuador, Malaysia, Indonesia and China; however, low acceptance rates (less than 60%) countries included Kuwait, Jordan, Italy, Russia, Poland, US and France [31]. Similar studies in the European region reported an acceptance rate of 60 to 80% [21, 22, 34, 35, 40–43]. In addition to those studies, an online study including 13,542 participants from 15 different countries reported China (20% hesitant) and France (60% hesitant) to be the most and least accepting countries for vaccination, respectively [44].

Similar studies have been conducted all over the world [34, 35, 40, 45–50]. The vaccination acceptance of vaccines is a multifactorial matter (Time, Location, Culture, Politics, Knowledge, Vaccine type, etc.), and investigation of the affecting factors will prove to be an asset in the future [51].

In addition to the mentioned factors, other thoughts questioning the vaccine, such as the speed of development and approval of these vaccines which are mostly produced by private companies also hinder the acceptance of the vaccines against this specific disease; COVID-19 [51, 52]. Furthermore, the widespread false information developed by anti-vaxxers and people against lockdown and other preventive methods is also another challenge that could affect the outcome of the vaccination campaign [53–55]. Although vaccination has been reported to be the most effective method for the prevention of certain diseases [56], there are still people (even professionals) who do not trust vaccines [57].

According to the results of the present study, approximately 30% of the participants were hesitant or against vaccination. Although this study was conducted before the mass-vaccination announcement, we can still compare the results with similar studies. Considering the fact that Iran was among the countries where vaccination was slow, and that the Iranian population has gone through multiple waves of the outbreak (this study was conducted during the fourth wave), the risk assessment and attitude of the population towards the disease would differ from other parts of the world. In a study performed in 15 different countries, the most common reason (57 to 80%) for vaccine hesitation was reported to be the unknown side-effects of the vaccines in all the countries. The second most common reason was reported to be doubt about the efficacy of vaccines (57% in Russia and 17% in Japan) [44].

In the present study, although 77% of the participants perceived the risk of infection, only 70% of them were willing to receive vaccines. Multiple studies report the perception of risk of infection to be a predictive factor in vaccine acceptance rate [14, 15, 29]. A study in Saudi Arabia concluded that people with a higher perception of the risk were 2.13 times more willing to receive vaccination, compare to those with lower perception [58]. This claim is in direction with similar studies in China, UK and South Korea [44].

In the present study, the only demographic characteristic that was seen to be effective in vaccine acceptance was education and surprisingly, the relationship was inverse, which was contrary to similar studies [59]. It would seem that better-educated people (with higher vaccine literacy) will have access to better and more valid sources for information and get to understand the unknown side effects, resulting in a lower acceptance rate among the better educated. The fact that vaccination started in most countries, before the announcement of the final results of vaccination trials, could also contribute to this hesitation.

In this study, participants under 50 reported 30% less acceptance rate compared to the participants above 50; although statistically insignificant. This can be supported by a similar study in Australia where the acceptance rate was reported to be lower in participants under 60 [59]. A study in Saudi Arabia found a higher acceptance rate among married participants, or those above 45 (1.15 and 2.79 times higher, respectively) [58]. Other studies also report higher chances of vaccine hesitation among the younger participants, further supporting our results [41, 60]. Elder individuals have a better risk perception which inspires better acceptance among them. Other demographic characteristics were found to be of insignificance to acceptance rate; however, other studies reported a relationship between female gender, lower economic and social status and lower education and lower acceptance rate [26]. The present study sheds light on the predictive status of vaccine literacy towards the acceptance rate. Previous studies did not find a relationship between health literacy and acceptance rate [61]. It is noteworthy that vaccine literacy is derived from health literacy [62], and health literacy facilitates the decision-making on receiving vaccines [63]. In addition to that, people with lower health literacy are more likely to believe in false information, hindering the decision-making regarding vaccination. Furthermore, lower health literacy is also a factor leading to mistrust of vaccination. In the present study, trusting in the health system was a predictive factor for vaccine acceptance. This finding is in accordance with similar studies [23, 24, 64].

A systematic review by Larson et al. concluded that mistrust of the health system is the second predicting factor for vaccine avoidance [17]. Generally, success in the vaccination campaign is highly affected by people’s trust in vaccine efficacy, safety, producers and government decisions [65].

Considering the facts presented above, strategies for the enhancement of societies’ trust in the vaccine are necessary.

### Limitations

The present study has many strengths and weaknesses. The large sample size was one of the strengths of this study. In addition to that, this was the first research regarding this matter in Iran. Selection bias can be considered the main limitation of this study. Considering the fact that sampling was done using online surveys, it is more likely that elder participants are not numerous enough. The same limitation applies to people with lower education.

## Conclusion

This study concludes that the vaccine acceptance rate in the Iranian population is 70%. The vaccine acceptance rate was higher than the other countries in the region. Education, attitude towards vaccination, vaccine literacy and trust in the health system are the main predictors of vaccine acceptance. Interventions are necessary in order to raise awareness with respect to the efficacy of vaccines, as well as trust promotion towards the success of vaccination.

### Implications

The results of this study offers information for policy-makers as well as researchers (as an initial study).

As knowledge of COVID-19 disease and attitude towards the vaccination were predictors of vaccine acceptance, therefore, the improvement of people’s information reduces people’s COVID-19 vaccine hesitancy, and this requires the efforts of many parties. Health care providers as one of the most trusted sources of information as well as media can play an effective role in enhancing trust among the population.

## Data Availability

The datasets used and/or analyzed during the current study are available from the corresponding author on reasonable request.

## Abbreviations

COVID-19: Corona Virus Disease of 2019
SPSS: Statistical Package for the Social Sciences
US: United States
UK: United Kingdom
CVI: Content validity indices
